# Association of Maternal and Child Health Center (*Posyandu*) Availability with Child Weight Status in Indonesia: A National Study

**DOI:** 10.3390/ijerph13030293

**Published:** 2016-03-07

**Authors:** Helen Andriani, Chu-Yung Liao, Hsien-Wen Kuo

**Affiliations:** 1 International Health Program, Institute of Public Health, National Yang Ming University, Taipei 112, Taiwan; helen_a9@yahoo.com; 2 Department of Early Childhood Educare, College of Health, Chung Chou University of Science and Technology, Changhua 510, Taiwan; cyliao@dragon.ccut.edu.tw; 3 Institute of Environmental and Occupational Health Sciences, National Yang-Ming University, Taipei 112, Taiwan; 4 School of Public Health, National Defense Medical Center, Taipei 112, Taiwan

**Keywords:** maternal, child, community-participation, primary health care, obesity, Indonesia

## Abstract

Little is known about the childhood obesity prevention and treatment practices of Maternal and Child Health services (*Posyandu*) in Indonesia or in other countries. The present study aims to assess the association of the availability of *Posyandu* with overweight and obesity in children of different household wealth levels. This was a secondary analysis of data collected in the 2013 Riskesdas (or Basic Health Research) survey, a cross-sectional study, representative population-based data. Height and weight, the availability of *Posyandu*, and basic characteristics of the study population were collected from parents with children aged 0 to 5 years (*n* = 63,237). Non-availability of *Posyandu* significantly raised the odds of being obese (OR = 1.13, 95% CI: 1.06–1.21) and did not show a significant relationship in the odds for overweight (OR = 0.99, 95% CI: 0.93–1.07). This relationship persisted after a full adjustment (OR = 1.16, 95% CI: 1.07–1.25 and OR = 1.04, 95% CI: 0.96–1.13, respectively). There was effect modification by household wealth, which was stronger for obese children. The availability of *Posyandu* has a protective association with childhood obesity in Indonesia. *Posyandu* services are well placed to play an important role in obesity prevention and treatment in early life.

## 1. Introduction

For quite some time, overweight and obesity were considered primarily problems of developed countries. However, with increasing incomes, urbanization, and changing lifestyles, the developing countries are facing the same issues [1]. Globally, the number of overweight children under the age of five in 2010 was estimated to be over 42 million. Close to 35 million of these are living in developing countries [2]. As a developing country, Indonesia is also facing a substantial increase in the numbers of overweight (including obese) children. While Indonesian government continues efforts to reduce hunger, that focus neglects the growing rate of overweight. In 1993, overweight prevalence among children under-five was 4.6%. This figure has increased very dramatically in 2007 and 2010, with the estimated prevalence of overweight was equal to 12.2% and 14.2%, respectively [3,4]. Overweight and obesity in children are associated with increased risk of hypertension, heart disease, diabetes mellitus, and sleep disturbances in adulthood [5,6].

The Indonesian government has focused on improving Maternal Child Health (MCH), by extending health services to urban and rural communities through the organization of volunteer-staffed Integrated Health Service Post *(Posyandu)*, following the international call of the Declaration of Alma-Ata (Kazakhstan) about Primary Health Care in 1978, convened by WHO and the United Nations Children’s Fund (UNICEF) [7]. *Posyandu* activity rapidly flourished throughout the nation: a jump up in the number of *Posyandu* from only 25,000 in 1985 to 244,382 in 1990. After that, the number of *Posyandu* in the country did not increase further, and may have even decreased [8]. Implementation of *Posyandu* requires intersectoral collaboration between the Department of Home Affairs and the Department of Health at the sub-district level [9].

The volunteers, who are called village health workers (*Kader*), have to be recruited and trained to recognize basic health care issues, such as nutrition, maternal and child health, family planning, immunization, and prevention of diarrhea. As part of the community, *Kader* would be much easier to deliver health programs because they are closer to the community compare to the public health officials. *Kader* usually are married women and members of the Family Welfare Movement. The *Kader* receive a week of training to carry out the *Posyandu* activities and a financial incentive for their work [10] Recruiting *Kader*, providing suitable venues and preparing for each monthly session are the shared responsibility of the local village community development committee, the Family Welfare Movement, and the village head. Programming and scheduling of sessions are coordinated by the health facility staff and the sub-district local government head, and health facility staff provides on-the-job training and supervise the *Kader* [11,12]. Historically in Indonesia, the presence of health volunteers and an active women’s organization at the village level have been credited with lowering fertility and improving child survival [13].

One *Posyandu* serves to approximately 50 children under 5 years of age, or its services are adjusted to the capability of the *Kader* and to local conditions, such as geographical conditions, distance between dwellings, number of households, *etc*. The *Posyandu* program is conducted every month in every village level. The operational of *Posyandu* is supported by medical doctor or midwife from sub district clinic and *Kader* or village volunteers. In terms of childhood obesity prevention, the *Kader* frequently undertake growth monitoring through weighing. In this way, the children’s weight gain can be monitored from one month to another. Therefore the children’s weight gain can be monitored from one month to another. In terms of childhood obesity treatment, in case of an increased trend of body weight or above the red line, the *Kader* are expected to give nutritional education or advice, make referrals to Public Health Centers, and address food supplements or feeding practices. *Posyandu* has been considered the most essential mechanism to enhance the nutrition improvement and toddler and baby mortality rate [14].

However, little is known about the childhood obesity prevention and treatment practices of *Posyandu* in Indonesia or in other countries. The limited number of existing studies to date have been studies exploring how participation of children in the *Posyandu* nutrition program improve children nutritional status [15] or a small scale qualitative study exploring the differences among the three *Posyandu* at different villages, how mother or father understood the growth chart, and the implications of gaining weight or not gaining [16]. Health status and service access differs substantially in particular between the rich and the poor [17]. The poor compared to the rich, have poorer health outcomes and is one of influencing factors contributing to health inequalities [18,19]. The rich-poor gap in child nutritional status, measured through the proportion of underweight children have been suggested including household expenditure [17]. We are unaware of any previous studies that comprehensively describe how the availability of *Posyandu* recently associate with overweight and obesity in children and whether such associations would change in different household wealth.

Therefore the aims of this paper were to: (1) examine the association of the availability of *Posyandu*, travel time to *Posyandu*, and travel cost to *Posyandu* with overweight and obesity in children, and (2) explore such associations in different household wealth.

## 2. Materials and Methods

### 2.1.Data Sources

This study involved a secondary analysis from the 2013 Riskesdas (or Basic Health Research) survey, a cross-sectional, nationally representative survey of the Indonesian population. The 2013 Riskesdas is the third survey conducted in Indonesia under the National Institute of Health Research and Development(NIHRD), Ministry of Health Republic of Indonesia. A two-stage, stratified cluster sampling approach was used for the selection of the survey sample. Two sampling frames were used for each stage. At the first stage, all 30,000 Primary Sampling Units included in the master list of census blocks were selected according to probability proportional to size (PPS) with the number of households Population Census in 2010. Two census blocks were selected according to PPS, while the size was the number of households in each districts based on the list of the 2010 Population Census. At the second stage, twenty five census buildings in each census blocks were selected using systematic random sampling. Finally one household in each census building was selected using random sampling. Sampling was conducted among a national sample of 150 sub census blocks in all 33 provinces with the total 497 districts/cities in Indonesia. A complete interview was obtained for 294,959 households from targeted 300,000 households (98.3 percent). The eligible children included all biological, step, or adopted children of the household head and spouse, as well as any children fostered to any adult in the household.

### 2.2. Measurement

The anthropometric measurements (height and weight) and information regarding the availability of *Posyandu* and basic characteristics of the study population were collected from parents with children aged 0 to 5 years in 2013. The trained interviewers (usually nurses) collected the height and weight measurements following accepted international standards. Standing height measures (for children over age two) and recumbent lengths (for younger children) were taken using a Multifunction brand (Brooklyn, NY, USA) stadiometer; measures of weight were taken using a Fesco (Brooklyn, NY, USA) digital weight scale, calibrated daily, including calibration across nurses who measure the height and weight. Both of these measuring instruments have been used in survey work in other countries and are suitable for field work given their portability, durability, and accuracy. Children who were too young or not able to stand on their own were held by a parent and weighted (after the scale had been adjusted to zero with just the parent alone on the scale). 

Height and weight were used to calculate Body Mass Index (BMI). BMI z-scores were determined for each child based on the 2006 WHO Child Growth Standards for children under five years old, age and sex specific. Underweight was defined as BMI z-score ≤ −2 SD. Healthy weight was defined as −2 SD < BMI z-score < 2 SD. Overweight was defined as 2 SD ≤ BMI z-score < 3 SD. Obese was defined as BMI z-score ≥ 3 SD [20,21].

There were 82,666 children under five years old in 2013. Of those, a total of 11,009 (13.3%) children with missing data on height and weight had to be eliminated from the sample. Children classified as underweight (8420 children or 10.2%) according to WHO were also excluded, leaving healthy weight, overweight, and obese status for the analysis. The final sample included 63,237 children. For the availability of *Posyandu*, data were collected through proxy interviews (usually a mother), the question was “Do you know if there are health facilites available nearby, including *Posyandu*? (Available/not available).” Nearby means at least one health facility, including *Posyandu* was located in the same or different village where the household was located. If the respondent answered “available”, then the next questions such as “How long does it take to visit the nearest health facilities, including *Posyandu*?” and “How much does it cost to visit the nearest health facilities, including *Posyandu*?” were asked. Travel time to *Posyandu* was categorized into: ≤15 min, >15 min, and not available. Travel cost to *Posyandu* (in Rupiah) was categorized into: <5000 Rupiah, ≥5,000 Rupiah, and not available. The outcome variable was child weight status, measured as categorical (healthy weight, overweight, and obese) variable. 

### 2.3. Potential Covariates and Effect Measure Modifier

We considered the following as covariates for child weight status: child’s gender (boy and girl), breastfeeding (no and yes), father’s education (none, elementary, junior high school, senior high school, and post-graduate), parental BMI (both parents <25 kg/m^2^, only mother ≥25 kg/m^2^, only father ≥25 kg/m^2^, and both parents ≥25 kg/m^2^), household wealth (poorer and wealthier), and residence (urban and rural). Poorer represents the lowest fifth of the data. Wealthier represents the second fifth to the highest fifth of the data. These self-reported covariates, collected through interviews, allowed for the control of variables that might influence child weight status. Household wealth was an effect modifier of interest.

### 2.4. Statistical Analyses

We performed all statistical analyses using SPSS 20.0 for Windows (IBM, Armonk, NY, USA). The variables in the study were evaluated using descriptive statistics including chi-square as appropriate. A multinomial logistic regression model, with healthy weight as a reference category controlled for covariates estimated the odds ratios (ORs) and 95% confidence intervals of overweight and obese. Data has been analyzed by adjusting the sampling weight for survey analysis. We set the statistical threshold for significance at 0.05. 

### 2.5. Ethical Considerations

The survey and its procedures were properly reviewed and approved by IRBs (LB.02.01/5.2/KE.006/2013) in Indonesia, at the Health Research Ethics Committee—University of Indonesia, Hasanuddin University, the University of Airlangga, and The National Institute of Health Research and Development (NIHRD), Ministry of Health Republic of Indonesia.

## 3. Results

### 3.1. Univariate Analysis for Children Weight Status and the Availability of Posyandu

Table 1 showed the availability of *Posyandu*, travel time to *Posyandu*, travel cost to *Posyandu*, and basic characteristics of the study population across categories of children weight status. We found the percentage of healthy weight, overweight, and obese children to be 76.4%, 6.0%, and 5.9%, respectively. Among the overweight or obese children, majority of them lived in the areas where a *Posyandu* existed, spent ≤15 min and <5000 Rupiah on travels, were boys, were breast-fed, had higher educated father, had healthy weight parents, or were wealthier.

### 3.2. The Association of the Availability of Posyandu, Travel Time to Posyandu, and Travel Cost to Posyandu with Overweight and Obese in Children

The association of the availability of *Posyandu* with child weight status was estimated using multinomial logistic regression model (Table 2). Non-availability of *Posyandu* significantly raised the odds of being obese (OR = 1.13, 95% CI: 1.06–1.21). This finding was confirmed by more objective measurement such as travel time to *Posyandu* (OR = 1.14, 95% CI: 1.06–1.22) and travel cost to *Posyandu* (OR = 1.21, 95% CI: 1.13–1.30). High travel cost (≥5000 Rupiah) to *Posyandu* significantly raised the odds of being overweight (OR = 1.15, 95% CI: 1.06–1.26) and obese (OR = 1.33, 95% CI: 1.22–1.44). After adjusting for the covariates, compared to the availability of *Posyandu*, non-availability of *Posyandu* showed a significant 1.16-fold increased odds of obesity (adjusted OR = 1.16, 95% CI: 1.07–1.25). This was confirmed by travel time to *Posyandu* (adjusted OR = 1.18, 95% CI: 1.09–1.27) and travel cost to *Posyandu* (adjusted OR = 1.24, 95% CI: 1.14–1.35). Compared to short travel time (≤15 min) to *Posyandu*, long travel time (>15 min) to *Posyandu* showed a significant 1.24-fold increased odds of obesity (adjusted OR = 1.24, 95% CI: 1.06–1.45). Compared to low travel cost (<5000 Rupiah) to *Posyandu*, high travel cost (≥5000 Rupiah) to *Posyandu* showed a significant 1.13-fold increased odds of overweight (adjusted OR = 1.13, 95% CI: 1.02–1.24) and 1.32-fold increased odds of obesity (adjusted OR = 1.32, 95% CI: 1.20–1.46).

### 3.3. The Association of the Availability of Posyandu, Travel Time to Posyandu, and Travel Cost to Posyandu with Overweight and Obese in Children in Different Household Wealth

From the stratified analyses in Table 3, compared to children who lived in the areas where a *Posyandu* existed, the odds ratio of being obese was statistically significantly higher among children from wealthier families (OR = 1.14, 95% CI: 1.06–1.24) who lived in the areas where a *Posyandu* did not exist. After adjusting for the covariates using multinomial logistic regression model, children from wealthier families (adjusted OR = 1.17, 95% CI: 1.07–1.27) who lived in the areas where a *Posyandu* did not exist were associated with higher odds of being obese. This was confirmed by travel time to *Posyandu* (adjusted OR = 1.17, 95% CI: 1.08–1.28) and travel cost to *Posyandu* (adjusted OR = 1.27, 95% CI: 1.16–1.39).

## 4. Discussion

Primary health care is practical, scientificially sound, socially acceptable health care made accessible and affordable at the country and community levels. The Alma-Ata Declaration, emphasises that primary health care should be accessible to all, and that it should involve everyone [7]. Using nationally representative population-based data concerning overweight and obesity in children in Indonesia, we pioneered an exploration of the associations of the availability of *Posyandu*, travel time to *Posyandu*, and travel cost to *Posyandu* with overweight and obesity in children of different household wealth levels. Our findings suggested that children who lived in areas without a *Posyandu* were more likely to be obese. 

Having a Posyandu more than 15 min away, or having to spend more than 5000 Rupiah on travel was associated with a greater risk of obesity than having no Posyandu. The reason for this is likely related to accessibility issues. Even if mothers were aware a Posyandu was nearby, they did not always access the services, perhaps due to geographic barriers (e.g., travel constraints) and economic barriers (e.g., travel expenses). This situation can impede seeking care. The reluctancy of visiting a *Posyandu* can in turn raise the risk of obesity. The rich-poor gaps in health status and service access remain crucial issues in Indonesia [17]. Household wealth was found to be an effect modifier of the relation between the availability of *Posyandu*, travel time to *Posyandu*, travel cost to *Posyandu* and child weight status. Non-availability of *Posyandu* and higher travel costs (≥5000 Rupiah) showed significant associations with the odds of being obese among wealthier families; longer travel time (>15 min) showed no such association. Wealthier parents have good jobs and typically want to provide their children a ”modern” lifestyle, providing plenty of modern recreational facilities, such as TVs and computers. These parents often give pocket money to their children, who then use the money to buy snacks. The results are a higher consumption high-calorie food and an avoidance of physically tough tasks. For these reasons, childhood overweight and obesity are more widespread in wealthier families [22]. According to our results, the risk of obesity in children of wealthier families living in communities without *Posyandu* was higher than that in children of poorer families. Some possible reasons are our limited sample size (*N* = 12,620) and factors characteristic in the poorer families, such as insufficient knowledge, inaccessible resources, and limited time. Overall, the prevalence of obesity in children was lower in the poorer.

In our study, we found that higher travel costs (≥5000 Rupiah) showed a significant association with the odds of being obese among wealthier families. For poorer families, longer travel time showed a significant association with the odds of being obese. These association was not particularly strong, and these variables were unable to explain much of the observed differences between households of different wealth levels.

Previous studies [23,24,25,26,27] have reported that outcomes of clinical interventions for pediatric obesity in primary care settings are variable. Some interventions promote changes in diet, physical activity, or television viewing but do not achieve reductions in BMI [25,26]. Others have favorable, although small, effects on BMI [23,24,27]. MCH centers are often unfeasible because of barriers associated with accessibility, transportation, and cost [28,29].

Our study had a number of limitations. BMI as a measure of weight status, given its consistently high specificity and good correlations with more direct measures of adiposity compared to other antropometric measurements, is generally useful in pediatric practice [30,31]. However, BMI is likely to introduce misclassification problems and an estimation bias for the effects related to the relationship between the availability of *Posyandu* and child weight status. Another limitation is the likely existence of other unmeasured variables that contribute to child weight status, such as the provision of infant feeding advice relevant to obesity prevention including best practice formula feeding and parental feeding behaviors as well as promoting active play and limiting sedentary behavior. The impact of variables such as these deserves further research. The primary limitation of the cross-sectional study design is the lack of evidence of a temporal relationship between exposure and outcome. A longitudinal design would provide a better picture about the impact of the availability of *Posyandu* on childhood obesity. There were no problems reported in understanding what information was requested for travel time and cost to *Posyandu*. However some respondents, particularly those who used private cars or motorcycles as transportation, might have lacked knowledge or obtained unclear information about the exact travel time and costs. To account for expenses, these participants needed to consider the distance driven, fuel costs, and other running costs of the car. Some respondents likely lacked the information to perform such accounting. Public transportation is easier to account for due to clear ticket costs. *Posyandu* has been considered as the most essential services at the village level to enhance the nutrition improvement of maternal and child health [14]. Eventhough mothers are encouraged to take their young children (five-year-olds and below) to the *Posyandu* instead of other health facilities, it is also possible that some mothers took their children to another health facility for any reason and could have resulted in an underestimation of the association between the availability of *Posyandu* and obesity in children. Our study did not emphasize the variance or weights of subpopulations such as the Graubard and Korn in our complex sample design [32]. When some sampled PSUs have no observations in the subset of interest, these assumptions are violated. The strength of our study was the dataset using a rigorous sampling design to select a large sample of a representative group of children. The number of participants included in this study was larger than those of other studies. Moreover, our measurement methods employed by trained interviewers (usually nurses) for height and weight are more accurate than self-reported measurements.The current scientific research on childhood obesity prevention and treatment practices of *Posyandu* in Indonesia is insufficient. This study adds to the limited information of the existing studies on the *Posyandu* nutrition program in Indonesia.

## 5. Conclusions

Our results suggest that the availability of *Posyandu* has a protective association with childhood obesity in Indonesia. Household wealth modified this association. Promoting healthy weight gain fits well with the *Posyandu* role. Solutions to improving obesity prevention and treatment practices may include further developing *Kader* counseling and behavior change skills, such as reflective listening, motivational interviewing and goal setting to enable them in raising the issue of weight in a sensitive and nonjudgmental manner in obesity prevention advice with parents’ roles in promoting optimal growth and development, specifically using delivery methods that overcome travel time and expense. The *Kader* should make continuous efforts to encourage the mothers to take their children to *Posyandu* regularly. With this additional support, *Posyandu* services are well placed to play an important role in obesity prevention and treatment in early life.

## Figures and Tables

**Table 1:** The availability of *Posyandu*, travel time to *Posyandu*, travel cost to *Posyandu*, and basic characteristics of the study population across categories of children weight status.

Variables	Categories	Total (*N* = 63,237)	Children Weight Status		
			Healthy Weight *n* (%) (*N* = 54,755)	Overweight *n* (%) (*N* = 4271)	Obese *n* (%) (*N* = 4211)
The Availability of *Posyandu*	Available	44,597 (70.5)	38,709 (70.7)	3020 (70.7)	2868 (68.1)
	Not Available	18,640 (29.5)	16,046 (29.3)	1251 (29.3)	1343 (31.9)
Travel Time to *Posyandu*	≤15 min	41,204 (65.2)	35,785 (65.4)	2787 (65.3)	2632 (62.5)
	>15 min	3393 (5.4)	2924 (5.3)	233 (5.5)	236 (5.6)
	Not Available	18,640 (29.5)	16,046 (29.3)	1251 (29.3)	1343 (31.9)
Travel Cost to *Posyandu*	<5000 Rupiah	34,659 (54.8)	30,278 (55.3)	2286 (53.5)	2095 (49.8)
	≥5000 Rupiah	9938 (15.7)	8431 (15.4)	734 (17.2)	773 (18.4)
	Not Available	18,640 (29.5)	16,046 (29.3)	1251 (29.3)	1343 (31.9)
Child’s Gender	Boys	31,640 (50.0)	27,243 (49.8)	2221 (52.0)	2176 (51.7)
	Girls	31,597 (50.0)	27,512 (50.2)	2050 (48.0)	2035 (48.3)
Breastfeeding	No	3580 (5.7)	1408 (2.6)	1226 (28.7)	946 (22.5)
	Yes	59,657 (94.3)	53,347 (97.4)	3045 (71.3)	3265 (77.5)
Father’s Education	None	6049 (11.6)	5290 (11.8)	354 (9.9)	405 (11.3)
	Elementary	12,669 (24.3)	10,953 (24.3)	888 (24.7)	828 (23.0)
	Junior High	10,552 (20.2)	9069 (20.1)	705 (19.6)	778 (21.6)
	Senior High	17,368 (33.3)	14,989 (33.3)	1218 (33.9)	1161 (32.3)
	Post-graduate	5553 (10.6)	4707 (10.5)	423 (11.8)	423 (11.8)
Parental BMI (kg/m^2^)	Both parents <25	26,326 (55.9)	22,723 (58.8)	1751 (41.2)	1852 (44.0)
	Only mother ≥25	9821 (20.8)	8039 (20.8)	1158 (27.2)	624 (14.8)
	Only father ≥25	5966 (12.7)	4752 (12.3)	815 (19.2)	399 (9.5)
	Both parents ≥25	5009 (10.6)	3146 (8.1)	530 (12.5)	1333 (31.7)
Household Wealth	Poorer	12,620 (20.0)	11,050 (20.2)	781 (18.3)	789 (18.7)
	Wealthier	50,617 (80.0)	43,705 (79.8)	3490 (81.7)	3422 (81.3)
Residence	Urban	28,809 (45.6)	24,903 (45.5)	1981 (46.4)	1925 (45.7)
	Rural	34,428 (54.4)	29,825 (54.5)	2290 (53.6)	2286 (54.3)

**Table 2:** Odds ratio and adjusted odds ratio (95% CI) using multinomial logistic regression model for overweight or obese compared to healthy weight according to the availability of *Posyandu*, travel time to *Posyandu*, and travel cost to *Posyandu.*

Variables	Categories	Overweight ^†^		Obese ^†^	
		cOR (95% CI)	aOR ^a^ (95% CI)	cOR (95% CI)	aOR ^a^ (95% CI)
The Availability of *Posyandu*	Available	1	1	1	1
	Not Available	0.99 (0.93–1.07)	1.04 (0.96–1.13)	1.13 ** (1.06–1.21)	1.16 ** (1.07–1.25)
Travel Time to *Posyandu*	≤15 min	1	1	1	1
	>15 min	1.02 (0.89–1.18)	1.16 (0.99–1.36)	1.10 (0.96–1.26)	1.24 ** (1.06–1.45)
	Not Available	1.01 (0.93–1.07)	1.06 (0.98–1.15)	1.14 ** (1.06–1.22)	1.18 ** (1.09–1.27)
Travel Cost to *Posyandu*	<5000 Rupiah	1	1	1	1
	≥5000 Rupiah	1.15 ** (1.06–1.26)	1.13 * (1.02–1.24)	1.33 ** (1.22–1.44)	1.32 ** (1.20–1.46)
	Not Available	1.03 (0.96–1.11)	1.07 (0.99–1.17)	1.21 ** (1.13–1.30)	1.24 ** (1.14–1.35)

Notes: cOR = Crude Odds Ratio; aOR = Adjusted Odds Ratio; CI = Confidence Interval; **^†^** Healthy weight is the reference category; * *p* < 0.05; ** *p* < 0.01; ^a^ adjusted for breastfeeding, father’s education, parental BMI, and household wealth.

**Table 3:** Odds ratio and adjusted odds ratio (95% CI) using multinomial logistic regression model for overweight or obese compared to healthy weight according to the availability of *Posyandu*, travel time to *Posyandu*, and travel cost to *Posyandu*, stratified by household wealth

**Variables**	**Categories**	**Poorer (*N* = 12,620)**			
		**Overweight ^†^**		**Obese ^†^**	
		**cOR**	**aOR ^a^ (95% CI)**	**cOR**	**aOR ^a^ (95% CI)**
The Availability of *Posyandu*	Available	1	1	1	1
	Not Available	1.01 (0.87–1.17)	0.97 (0.81–1.15)	1.14 (0.98–1.32)	1.08 (0.91–1.27)
Travel Time to *Posyandu*	≤15 min	1	1	1	1
	>15 min	1.28 * (1.02–1.60)	1.46 ** (1.12–1.90)	1.30 * (1.04–1.64)	1.50 ** (1.16–1.93)
	Not Available	1.06 (0.91–1.24)	1.05 (0.87–1.26)	1.20 * (1.03–1.41)	1.17 (0.98–1.40)
Travel Cost to *Posyandu*	<5000 Rupiah	1	1	1	1
	≥5000 Rupiah	0.99 (0.78–1.26)	0.82 (0.62–1.08)	1.33 * (1.06–1.66)	1.19 (0.93–1.53)
	Not Available	1.01 (0.86–1.17)	0.93 (0.77–1.12)	1.21 * (1.04–1.41)	1.12 (0.94–1.34)
**Variables**	**Categories**	**Wealthier (*N* = 50,617)**			
		**Overweight ^†^**		**Obese ^†^**	
		**cOR**	**aOR ^a^ (95% CI)**	**cOR**	**aOR ^a^ (95% CI)**
The Availability of *Posyandu*	Available	1	1	1	1
	Not Available	1.01 (0.94–1.10)	1.05 (0.96–1.15)	1.14 ** (1.06–1.24)	1.17 ** (1.07–1.27)
Travel Time to *Posyandu*	<15 min	1	1	1	1
	>15 min	0.94 (0.78–1.13)	0.97 (0.78–1.20)	1.05 (0.87–1.26)	1.07 (0.87–1.31)
	Not Available	1.01 (0.94–1.09)	1.05 (0.96–1.15)	1.15 ** (1.06–1.24)	1.17 ** (1.08–1.28)
Travel Cost to *Posyandu*	<5000 Rupiah	1	1	1	1
	≥5000 Rupiah	1.18 ** (1.07–1.29)	1.19 ** (1.07–1.32)	1.32 ** (1.20–1.45)	1.36 ** (1.23–1.51)
	Not Available	1.05 (0.97–1.14)	1.10 * (1.01–1.21)	1.23 ** (1.13–1.33)	1.27 ** (1.16–1.39)

Notes: cOR = Crude Odds Ratio; aOR = Adjusted Odds Ratio; CI = Confidence Interval; ^†^ Healthy weight is the reference category; * *p* <0.05; ** *p* < 0.01; ^a^ adjusted for breastfeeding, father’s education, and parental BMI.
